# The Plant Negative-Sense RNA Virosphere: Virus Discovery Through New Eyes

**DOI:** 10.3389/fmicb.2020.588427

**Published:** 2020-09-16

**Authors:** Nicolás Bejerman, Humberto Debat, Ralf G. Dietzgen

**Affiliations:** ^1^Instituto de Patología Vegetal – Centro de Investigaciones Agropecuarias – Instituto Nacional de Tecnología Agropecuaria, Córdoba, Argentina; ^2^Consejo Nacional de Investigaciones Científicas y Técnicas, Unidad de Fitopatología y Modelización Agrícola, Buenos Aires, Argentina; ^3^Queensland Alliance for Agriculture and Food Innovation, The University of Queensland, St. Lucia, QLD, Australia

**Keywords:** HTS, virus discovery, plant NSR viruses, virosphere, bioinformactics

## Abstract

The use of high-throughput sequencing (HTS) for virus diagnostics, as well as the importance of this technology as a valuable tool for discovery of novel viruses has been extensively investigated. In this review, we consider the application of HTS approaches to uncover novel plant viruses with a focus on the negative-sense, single-stranded RNA virosphere. Plant viruses with negative-sense and ambisense RNA (NSR) genomes belong to several taxonomic families, including *Rhabdoviridae, Aspiviridae, Fimoviridae, Tospoviridae*, and *Phenuiviridae.* They include both emergent pathogens that infect a wide range of plant species, and potential endophytes which appear not to induce any visible symptoms. As a consequence of biased sampling based on a narrow focus on crops with disease symptoms, the number of NSR plant viruses identified so far represents only a fraction of this type of viruses present in the virosphere. Detection and molecular characterization of NSR viruses has often been challenging, but the widespread implementation of HTS has facilitated not only the identification but also the characterization of the genomic sequences of at least 70 NSR plant viruses in the last 7 years. Moreover, continuing advances in HTS technologies and bioinformatic pipelines, concomitant with a significant cost reduction has led to its use as a routine method of choice, supporting the foundations of a diverse array of novel applications such as quarantine analysis of traded plant materials and genetic resources, virus detection in insect vectors, analysis of virus communities in individual plants, and assessment of virus evolution through ecogenomics, among others. The insights from these advancements are shedding new light on the extensive diversity of NSR plant viruses and their complex evolution, and provide an essential framework for improved taxonomic classification of plant NSR viruses as part of the realm *Riboviria*. Thus, HTS-based methods for virus discovery, our ‘new eyes,’ are unraveling in real time the richness and magnitude of the plant RNA virosphere.

## Introduction

Viruses are the most numerous biological entities on Earth, but the number of reported and formally described virus species, the known virosphere, is exiguous. This underestimated global virus landscape has led to a distorted view of virus diversity and function. Some aspects of virus biology including their small size, rapid rate of evolution, or lack of universally conserved viral genetic markers are likely the major cause for the lack of knowledge of most of the viruses present on our planet (Koonin et al., 2015; Zhang et al., 2019). Thus, as we explore the virosphere, it becomes evident that only a tiny proportion (about 1% according to some estimates) has been characterized, with a major bias against the identification of the most divergent genomes (Zhang et al., 2018).

High-throughput sequencing (HTS) technology, also referred to as next generation sequencing, has revolutionized the nucleic acid characterization process since it allows the parallel sequencing of millions of nucleotides in a short period of time at a very high redundancy (depth of sequencing) without any *a priori* knowledge. Thus, when combined with specific bioinformatics tools, HTS provides a powerful, efficient and economical alternative that has enabled not only the untargeted detection of both known and unknown viruses that inhabit a particular organism or environment but also a rapid characterization of their genomes (Massart et al., 2017; Villamor et al., 2019). Thus, the steady increase in the adoption of HTS in the past decade has accelerated virus discovery of both wild and cultivated plant species, which led to the advancement of our knowledge about the diversity of viruses in nature.

High-throughput sequencing is also widely used to advance the molecular characterization not only of those viruses which have a poorly characterized genome, but also of preserved historic virus isolates which had been subjected to studies of their biological properties some decades ago. For instance, sterile stunt disease of maize, characterized by severe stunting and top necrosis of susceptible maize genotypes, was first reported in 1977 in Australia, but the genome of its etiological agent, maize sterile stunt virus was only characterized by HTS and recently reported (Dietzgen and Higgins, 2019). Similarly, sowthistle yellow vein virus (SYVV) was recently rediscovered and its genome characterized after a hiatus of over 30 years following pioneering virus and vector biology research (Stenger et al., 2020).

In spite of the many advantages of HTS, there are some limitations and constraints that should be considered when using this platform for virus discovery. Given the high sensitivity of HTS, this technology may detect contaminant viral sequences or viruses that may not be actually replicating in the sampled plant tissue where they were found (Blawid et al., 2017). In this context it is worth emphasizing the importance of traditional wet bench virology experiments to complement viral HTS data where possible, such as virus isolation and transmission experiments, *in situ* virus particle detection by electron microscopy or immunoassays, to name a few.

Most of the plant viruses described so far, whose nucleotide sequences are available in public databases, were discovered in exemplars of cultivated plant species that showed conspicuous disease symptoms, limiting our view of viral diversity (Wren et al., 2006). However, the steady increase in the last few years of metagenomic studies to characterize the viromes of wild plant species, many of which did not show any visible symptoms, led to the identification of many new viruses. Moreover, several recent reports provide evidence of viruses which appear to be essentially cryptic, identified from cultivated plants (Bernardo et al., 2018; Schoelz and Stewart, 2018; Susi et al., 2019; Ma et al., 2020). Nevertheless, for most of the newly discovered viruses, subsequent studies to characterize their biological properties, such as symptoms in different hosts, and potential vectors, among others, in both cultivated and wild plant species are scarce, and likely will be neglected due to lack of economic significance or unavailability of preserved samples. The huge gap between virus discovery and biological characterization of new viruses, is due to the latter requiring time-consuming research efforts. Therefore, we are advancing into a scenario where the classification of most novel viruses will be based *only* on their genomic sequences which constitutes a paradigm shift in their taxonomical classification (Simmonds et al., 2017; Kuhn et al., 2019), thus representing a major challenge for virologists and the International Committee on Taxonomy of Viruses (ICTV).

Although many pipelines are available for plant virus discovery through HTS, all share a common backbone (Villamor et al., 2019). Various HTS technologies are available commercially, but Illumina short read shotgun sequencing platforms are the most popular choice and most widely used for HTS of viruses because of their high throughput, low error rate and high cost effectiveness among currently available HTS platforms (Villamor et al., 2019). Four main classes of nucleic acids have been targeted as templates for HTS, (*i*) total plant RNA extracts, usually with a ribosomal depletion step (*ii*) virion-associated nucleic acids (VANA) extracted from purified viral particles, (*iii*) double-stranded RNA (dsRNA), enriched through cellulose chromatography or monoclonal antibody pull-down, and (*iv*) small interfering RNAs (siRNAs) (Roossinck et al., 2015; Wu et al., 2015; Blouin et al., 2016). These different strategies are characterized by diverse caveats. For instance, VANA is inefficient in detecting viruses that lack virions, or siRNA-based protocols are not as reliable for complete genome assembly of novel viruses. Sequencing of siRNAs and total RNA are the two approaches most generically applicable to viruses with different genome types and replication strategies, and can be relatively easily integrated into workflows of diagnostic laboratories (Pecman et al., 2017).

Recent technological advances have led to the appreciation of exciting novel aspects of negative-sense and ambisense RNA (NSR) viruses, as recently reviewed by German et al. (2020). Plant NSR viruses are considered emerging viral pathogens (German et al., 2020). In light of the growing interest in these viruses, in this review we delve into the application of HTS approaches to uncover the abundance and diversity of the negative-sense, single-stranded RNA virosphere associated with plants.

## High-Throughput Sequencing to Uncover the Negative-Sense, Single-Stranded RNA Virosphere of Plants

Hundreds of plant viruses have been characterized by HTS in the last few years (Blawid et al., 2017; Villamor et al., 2019), and most of them have positive-sense RNA genomes. On the other hand, RNA viruses with negative-sense and ambisense genomes discovered through HTS represent only a small fraction of the total number of viruses discovered, thus representing a tiny fraction of the plant virosphere. For example, the characterization of the virome of different *Solanum* species resulted in the identification of viruses belonging to 20 different families, but only one was a NSR virus (Ma et al., 2020). In addition, the characterization of a tomato virome resulted in the identification of 22 viruses belonging to 12 genera, but only three genomes corresponded to NSR viruses (Xu et al., 2017). Moreover, the characterization of a papaya virome resulted in the identification of 52 viruses, but only one was a NSR virus (Alcalá-Briseño et al., 2020). It is worth mentioning that typically, virome studies based on HTS generate a significant amount of sequence contigs that lack detectable homology to both the sampled host and any microorganism. It has been suggested that a fraction of these sequences may correspond to viral ‘dark’ matter, which may imply that many deeply divergent viruses, or viruses lacking common ancestry or similarity with known virus families, remain to be discovered; this may not happen until better frameworks are implemented to identify viral sequences regardless of their sequence similarity to known viruses (Obbard et al., 2020). Current virome analyses usually rely on sequence similarity searches to identify virus-like sequences through inferred homology. This approach limits the identification of new viruses that can be discovered through traditional empirical search algorithms such as BLAST using identity thresholds of target sequences to genomes, genes, proteins or protein motifs of known viruses (Obbard et al., 2020). As a consequence, in contrast to the more straightforward hypothesis that plant NSR viruses are relatively rare, it is plausible that NSR virus sequences may have been overlooked due to extreme divergence from known viruses, thus providing an alternative reason why so few NSR viruses have been identified when the viromes of different plant hosts are characterized. Nevertheless, as novel bioinformatics tools are developed to increase the sensitivity of similarity search algorithms and more refined *ab initio* probabilistic methods are implemented, such as hidden Markov models that incorporate position-specific information into the alignment process of a group of highly divergent, evolutionarily related sequences and use these profiles to identify virus sequences (Skewes-Cox et al., 2014) or methods that rely on support vector machines (Liao and Noble, 2003), the identification of novel, more divergent viruses will likely become possible. This may result in an increased number of identified NSR viruses, which will allow us to deepen our understanding about the evolution and diversity of NSR viruses. This is clearly illustrated by the discovery of two NSR viruses associated with apple rubbery wood disease. The initial attempt to determine by NGS if any virus was associated with the disease was unsuccessful (Jakovljevic et al., 2016). However, the NGS data was later reanalyzed in depth using a bioinformatics approach focused on viral conserved protein motifs that resulted in the identification and genome assembly of two novel NSR viruses (Rott et al., 2018).

The use of HTS technologies has not only allowed the identification and characterization of novel NSR viruses in several plant hosts (Table 1), but has also enabled the completion of genome sequences of NSR viruses for which biological properties and only partial genome fragments were known (Table 2). The two nucleic acid classes mainly targeted when using HTS to sequence NSR viruses are total RNA and siRNAs (Tables 1, 2). When Pecman et al. (2017) compared these approaches they found that both can be used to detect and identify a wide array of known plant viruses in the tested samples including orthotospoviruses. However, on this occasion a putative novel cytorhabdovirus genome could only be assembled *de novo* from the sequencing data generated from total RNA and not from the small RNA dataset, due to the low number of short reads in the latter (Pecman et al., 2017), thus the choice of nucleic acid types used in HTS may have an effect on the range of viruses that can be identified. However, a few novel plant rhabdoviruses including cytorhabdoviruses have been identified using small RNA as sequencing template (Table 1).

**TABLE 1:** Novel NSR viruses discovered using HTS.

Virus family/genus	Virus	Type of target/sequencing technology	References
*Aspiviridae/Ophiovirus*	Blueberry mosaic associated ophiovirus	Total nucleic acid/Illumina HiSeq	Thekke-Veetil et al. (2014)
*Phenuiviridae/Coguvirus*	Citrus concave gum-associated virus	Small RNAs/Illumina HiSeq; total RNA/Illumina HiSeq	Navarro et al. (2018a); Wright et al. (2018)
*Phenuiviridae/Coguvirus*	Citrus virus A	Small RNAs/Illumina Genome HiScan	Navarro et al. (2018b)
*Phenuiviridae/Coguvirus*	Watermelon crinkle leaf-associated virus 1	Small RNAs and total RNA/Illumina HiSeq	Xin et al. (2017)
*Phenuiviridae/Coguvirus*	Watermelon crinkle leaf-associated virus 2	Small RNAs and total RNA/Illumina HiSeq	Xin et al. (2017)
*Phenuiviridae/Coguvirus*	Grapevine associated cogu-like virus 1	Total RNA/Illumina HiSeq	Chiapello et al. (2020)
*Phenuiviridae/Coguvirus*	Grapevine associated cogu-like virus 2	Total RNA/Illumina HiSeq	Chiapello et al. (2020)
*Phenuiviridae/Coguvirus*	Grapevine associated cogu-like virus 3	Total RNA/Illumina HiSeq	Chiapello et al. (2020)
*Phenuiviridae/Coguvirus*	Grapevine associated cogu-like virus 4	Total RNA/Illumina NovaSeq	Bertazzon et al. (2020)
*Phenuiviridae/Rubodvirus*	Apple rubbery wood virus 1	Double-stranded RNA/Illumina HiSeq; total RNA/Illumina HiSeq	Rott et al. (2018); Wright et al. (2018)
*Phenuiviridae/Rubodvirus*	Apple rubbery wood virus 2	Double-stranded RNA/Illumina HiSeq; total RNA/Illumina HiSeq	Rott et al. (2018); Wright et al. (2018)
*Phenuiviridae/Rubodvirus*	Grapevine Muscat rose virus	Total nucleic acid/Illumina HiSeq	Diaz-Lara et al. (2019)
*Phenuiviridae/Rubodvirus*	Grapevine Garan dmak virus	Total nucleic acid/Illumina HiSeq	Diaz-Lara et al. (2019)
*Phenuiviridae/Tenuivirus*	Melon chlorotic spot virus	Small RNAs/Illumina HiSeq; total RNA/Illumina MiSeq	Lecoq et al. (2019); Gaafar et al. (2019a)
*Phenuiviridae/Tenuivirus*	Ramu stunt virus	Total RNA/Illumina HiSeq	Mollov et al. (2016)
*Phenuiviridae/Tenuivirus*	European wheat striate mosaic virus	Small and total RNA/Illumina HiSeq and NextSeq	Sõmera et al. (2020)
*Tospoviridae/Orthotospovirus*	Alstroemeria yellow spot virus	Total RNA/Illumina HiSeq	Hassani-Mehraban et al. (2019)
*Tospoviridae/Orthotospovirus*	Chili yellow ringspot virus	Total RNA/Illumina MiSeq	Zheng et al. (2020)
*Fimoviridae/Emaravirus*	Blackberry leaf mottle associated virus	Double-stranded RNA/Illumina and 454-Junior (Roche)	Hassan et al. (2017)
*Fimoviridae/Emaravirus*	Pigeonpea sterility mosaic virus 2	Double-stranded RNA/Illumina HiScan; small RNAs/Illumina GAIIx	Elbeaino et al. (2015); Kumar et al. (2017)
*Fimoviridae/Emaravirus*	Pistacia virus B	Total RNA/Illumina NextSeq	Buzkan et al. (2019)
*Fimoviridae/Emaravirus*	Redbud yellow ringspot-associated virus	Double-stranded RNA/Illumina	Di Bello et al. (2016)
*Fimoviridae/Emaravirus*	Ti ringspot-associated virus	Double-stranded RNA/Illumina HiSeq and MiSeq	Olmedo-Velarde et al. (2019)
*Fimoviridae/Emaravirus*	Actinidia chlorotic ringspot-associated virus	Small RNAs/Illumina Genome analyzer	Zheng et al. (2017)
*Fimoviridae/Emaravirus*	Jujube yellow mottle-associated virus	Small RNAs/BGISEQ and total RNA/Illumina HiSeq	Yang et al. (2019)
*Fimoviridae/Emaravirus*	Blue palo verde broom virus	Total RNA/Illumina HiSeq	Ilyas et al. (2018)
*Fimoviridae/Emaravirus*	Camellia japonica associated emaravirus 1	Total RNA/Illumina HiSeq	Peracchio et al. (2020)
*Fimoviridae/Emaravirus*	Camellia japonica associated emaravirus 2	Total RNA/Illumina HiSeq	Peracchio et al. (2020)
*Fimoviridae/Emaravirus*	Aspen mosaic-associated virus	Total RNA/Illumina HiSeq	von Bargen et al. (2020)
*Fimoviridae/Emaravirus*	Perilla mosaic virus	Total RNA/Illumina NovaSeq	Kubota et al. (2020)
*Fimoviridae/Emaravirus*	Lilac chlorotic ringspot-associated virus	Total RNA/Illumina HiSeq	Wang et al. (2020)
*Rhabdoviridae/Cytorhabdovirus*	Strawberry associated virus 1	Small RNAs/Illumina MiSeq; total RNA/Illumina HiSeq and PCR products/Ion Proton	Ding et al. (2019); Franova et al. (2019a)
*Rhabdoviridae/Cytorhabdovirus*	Tomato yellow mottle-associated virus	Small RNAs/Illumina HiSeq	Xu et al. (2017)
*Rhabdoviridae/Cytorhabdovirus*	Maize associated rhabdovirus	Total RNA/Illumina HiSeq	Willie and Stewart (2017)
*Rhabdoviridae/Cytorhabdovirus*	Colocasia bobone disease-associated virus	Total RNA/Illumina HiSeq	Higgins et al. (2016)
*Rhabdoviridae/Cytorhabdovirus*	Papaya virus E; bean-associated cytorhabdovirus; citrus-associated rhabdovirus	Total RNA/Illumina HiSeq; double stranded RNA/Illumina MiSeq; total RNA/Illumina HiSeq	Medina-Salguero et al. (2019); Alves-Freitas et al. (2019); Zhang et al. (2020)
*Rhabdoviridae/Cytorhabdovirus*	Cabbage cytorhabdovirus 1	Total RNA/Illumina MiSeq	Pecman et al. (2017)
*Rhabdoviridae/Cytorhabdovirus*	Rice stripe mosaic virus	Small RNAs/Illumina HiSeq	Yang et al. (2017)
*Rhabdoviridae/Cytorhabdovirus*	Wuhan insect virus 4	Total RNA/Illumina HiSeq	Li et al. (2015)
*Rhabdoviridae/Cytorhabdovirus*	Wuhan insect virus 5	Total RNA/Illumina HiSeq	Li et al. (2015)
*Rhabdoviridae/Cytorhabdovirus*	Wuhan insect virus 6	Total RNA/Illumina HiSeq	Li et al. (2015)
*Rhabdoviridae/Cytorhabdovirus*	Trifolium pratense virus A	Total RNA/Illumina HiSeq	Franova et al. (2019b)
*Rhabdoviridae/Cytorhabdovirus*	Trifolium pratense virus B	Total RNA/Illumina HiSeq	Franova et al. (2019b)
*Rhabdoviridae/Cytorhabdovirus*	Yerba mate chlorosis-associated virus	Small RNAs/Illumina HiSeq	Bejerman et al. (2017)
*Rhabdoviridae/Cytorhabdovirus*	Yerba mate virus A	Total RNA/Illumina HiSeq	Bejerman et al. (2020)
*Rhabdoviridae/Cytorhabdovirus*	Persimmon Virus A	Total RNA/Illumina Genome Analyzer	Ito et al. (2013)
*Rhabdoviridae/Cytorhabdovirus*	Trichosantes associated rhabdovirus 1	Total RNA/Illumina HiSeq	Goh et al. (2020)
*Rhabdoviridae/Cytorhabdovirus*	Cucurbit cytorhabdovirus 1	Total RNA/Illumina NovaSeq	Orfanidou et al. (2020)
*Rhabdoviridae/Betanucleorhabdovirus*	Alfalfa-associated nucleorhabdovirus	Total RNA/Illumina MiSeq	Gaafar et al. (2019b)
*Rhabdoviridae/Betanucleorhabdovirus*	Apple rootstock virus A	Total RNA/Illumina HiSeq	Baek et al. (2019)
*Rhabdoviridae/Alphanucleorhabdovirus*	Physostegia chlorotic mottle virus	Total RNA/Illumina MiSeq	Menzel et al. (2016); Gaafar et al. (2018)
*Rhabdoviridae/Alphanucleorhabdovirus*	Morogoro maize-associated virus	Total RNA/Illumina HiSeq	Read et al. (2019)
*Rhabdoviridae/Betanucleorhabdovirus*	Black currant associated rhabdovirus	Total RNA/Illumina NextSeq	Wu et al. (2018)
*Rhabdoviridae/Alphanucleorhabdovirus*	Wheat yellow striate virus	Total RNA/Illumina HiSeq	Liu et al. (2018)
*Rhabdoviridae/Betanucleorhabdovirus*	Green Sichuan pepper nucleorhabdovirus	Small RNAs and total RNA/Illumina HiSeq	Cao et al. (2019)
*Rhabdoviridae/Betanucleorhabdovirus*	Bird’s-foot trefoil-associated virus 1	Total RNA/Illumina HiSeq	Debat and Bejerman (2019)
*Rhabdoviridae/Alphanucleorhabdovirus*	Peach virus 1	Total RNA/Illumina HiSeq	Zhou et al. (2020)
*Rhabdoviridae/Betanucleorhabdovirus*	Cardamom vein clearing rhabdovirus 1	Small RNA/Illumina HiSeq	Bhat et al. (2020)
*Rhabdoviridae/Dichorhavirus*	Clerodendrum chlorotic spot virus	Total RNA/Illumina HiSeq	Ramos-González et al. (2018)
*Rhabdoviridae/Dichorhavirus*	Citrus leprosis virus N	Total RNA/Illumina HiSeq	Ramos-González et al. (2017)
*Rhabdoviridae/Dichorhavirus*	Citrus chlorotic spot virus	Total RNA/Illumina HiSeq	Chabi-Jesus et al. (2018)
*Rhabdoviridae/Varicosavirus*	Red clover-associated varicosavirus	Double stranded RNA/Illumina HiSeq	Koloniuk et al. (2018a)
*Rhabdoviridae/Varicosavirus*	Alopecurus myosuroides varicosavirus 1	Total RNA/454 (Roche) and Illumina HiSeq	Sabbadin et al. (2017)

**TABLE 2:** NSR viruses with partial genome sequence already know, whose genomes were characterized using HTS.

Virus family/genus	Virus	Type of target/sequencing technology	References
*Phenuiviridae/Tenuivirus*	Rice hoja blanca virus	Small RNAs/Illumina HiSeq	Jimenez et al. (2018)
*Tospoviridae/Orthotospovirus*	Groundnut chlorotic fan-spot virus	Total RNA/Illumina MiSeq	Chou et al. (2017)
*Tospoviridae/Orthotospovirus*	Chrysanthemum stem necrosis virus	Viral RNA from ribonucleocapsid/454 (Roche)	Dullemans et al. (2015)
*Tospoviridae/Orthotospovirus*	Mulberry vein banding associated virus	Small RNAs/Illumina Genome Analyzer	Meng et al. (2015)
*Tospoviridae/Orthotospovirus*	Alstroemeria necrotic streak virus	Total RNA/Illumina HiSeq	Gallo et al. (2018)
*Tospoviridae/Orthotospovirus*	Melon severe mosaic virus	Total RNA/Illumina NextSeq	Ciuffo et al. (2017)
*Tospoviridae/Orthotospovirus*	Zucchini lethal chlorosis virus	Viral RNA from ribonucleocapsid/Illumina HiSeq	Lima et al. (2016)
*Tospoviridae/Orthotospovirus*	Tomato chlorotic spot virus	Total RNA/Illumina HiSeq	Martínez et al. (2018); Adegbola et al. (2019), Fagundes Silva et al. (2019)
*Tospoviridae/Orthotospovirus*	Polygonum ringspot virus	Small RNAs	Margaria et al. (2014)
*Fimoviridae/Emaravirus*	Rose rosette virus	Total nucleic acid/Illumina HiSeq and 454-Junior (Roche)	Di Bello et al. (2015)
*Fimoviridae/Emaravirus*	Pigeonpea sterility mosaic virus 1	Double-stranded RNA/Illumina HiScan; small RNAs/Illumina GAIIx	Elbeaino et al. (2014); Kumar et al. (2017)
*Fimoviridae/Emaravirus*	Raspberry leaf blotch virus	Total RNA/Illumina MiSeq	Lu et al. (2015)
*Fimoviridae/Emaravirus*	European mountain ash ringspot-associated virus	Total RNA/Illumina HiSeq	von Bargen et al. (2019)
*Fimoviridae/Emaravirus*	High plains wheat mosaic virus	Partially purified virion RNA/Illumina MiSeq	Tatineni et al. (2014)
*Rhabdoviridae/Cytorhabdovirus*	Alfalfa dwarf virus	Small RNAs/Illumina HiSeq	Bejerman et al. (2015)
*Rhabdoviridae/Cytorhabdovirus*	Raspberry vein chlorosis virus	Total RNA/Illumina NextSeq	Jones et al. (2019)
*Rhabdoviridae/Cytorhabdovirus*	Barley yellow striate mosaic virus/maize sterile stunt virus	Small RNAs/Illumina HiSeq; viral enriched RNA/454 (Roche)	Yan et al. (2015); Dietzgen and Higgins (2019)
*Rhabdoviridae/Cytorhabdovirus*	Strawberry crinkle virus	Total RNA/Illumina HiSeq	Koloniuk et al. (2018b)
*Rhabdoviridae/Cytorhabdovirus*	Maize yellow striate virus	Viral enriched RNA/Illumina HiSeq	Maurino et al. (2018)
*Rhabdoviridae/Betanucleorhabdovirus*	Datura yellow vein virus	Total RNA/454 (Roche)	Dietzgen et al. (2015)
*Rhabdoviridae/Alphanucleorhabdovirus*	Maize mosaic virus	Total RNA/Illumina HiSeq	Martin and Whitfield (2019)
*Rhabdoviridae/Alphanucleorhabdovirus*	Maize Iranian mosaic virus	Total RNA/Illumina HiSeq	Ghorbani et al. (2018)
*Rhabdoviridae/Betanucleorhabdovirus*	Sowthistle yellow vein virus	Total RNA/Oxford Nanopore	Stenger et al. (2020)
*Rhabdoviridae/Dichorhavirus*	Coffee ringspot virus	Total RNA/ION Torrent	Ramalho et al. (2014)

Most plant NSR viruses are transmitted by arthropods in a persistent-circulative and propagative manner, thus they are adapted to infect both arthropod and plant cells. In fact, it has been suggested that plant NSR viruses may have originated from arthropod viruses that evolved to also infect plants (Whitfield et al., 2018; Dolja et al., 2020). Therefore, another potential source to discover novel plant-associated NSR viruses is the characterization of arthropod vector viromes. For instance, Li et al. (2015) performed deep transcriptome sequencing of 70 arthropod species that resulted in the identification of three novel cytorhabdoviruses (Wuhan insect viruses 4–6) associated with the mealy plum aphid (*Hyalopterus pruni*). Wuhan insect viruses 4–6 may be plant viruses based on their clear phylogenetic relationship with plant rhabdoviruses in the genus *Cytorhabdovirus* and that their genomes encode a P3 protein that most resembles plant rhabdovirus cell-to-cell movement proteins.

Since most NSR viruses replicate in both the plant host and the arthropod vector, the true virus host origins cannot be unambiguously discerned from the HTS data. Some of the newly discovered NSR viruses are highly divergent which makes it even more difficult to unequivocally determine if they are plant or plant-associated viruses, and it will require a significant amount of research to confirm their status, which is rarely carried out. This is exemplified by the recently discovered coguviruses and rubodviruses, where the host assignment based on their phylogenetic relationships is only preliminary. Thus, further biological studies will be required to determine if they are plant, plant-associated, or insect viruses (Navarro et al., 2018b; Diaz-Lara et al., 2019; Chiapello et al., 2020).

## Negative-Sense, Single-Stranded RNA Virus Diversity and Taxonomy

**FIGURE 1:**
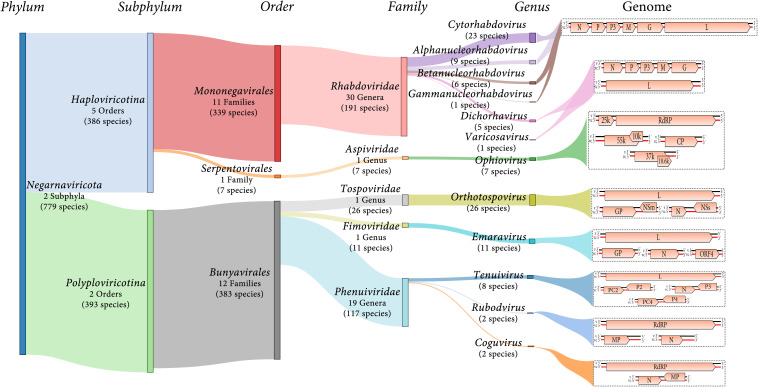
Diagram showing an overview of plant NSR virus taxonomy and schematic genome graphs depicting organization and gene products of representative members of each taxon. The predicted coding sequences are shown in orange arrowed boxes, virion-sense RNA (v) is depicted in black, while virion-complementary sense RNA (vc) is depicted in red. The number of taxonomic categories included in each taxon are indicated. Species number indicate 2019 ICTV-accepted virus species corresponding to each taxonomic rank.

Negative-sense and ambisense RNA viruses belong to the phylum *Negarnaviricota*, which includes members characterized by (*i*) negative-sense or ambisense single-stranded unsegmented or segmented genomes, (*ii*) the presence or absence of a lipid membrane enveloping the capsid, and (*iii*) a diverse host range (Diaz-Lara et al., 2019; Koonin et al., 2020; Kuhn et al., 2020). This phylum contains major groups of pathogenic viruses and our current knowledge of these viruses is strongly biased toward agents with special importance for human and animal health, such as influenza virus (*Orthomyxoviridae*), Zaire ebolavirus (*Filoviridae*) or Crimean-Congo hemorrhagic fever virus (*Nairovirus*). Most of the genera contain NSR viruses that infect vertebrates and only about 10% contain phytoviruses (Käfer et al., 2019) (Figure 1).

Most of the NSR viruses are divided into two large lineages based on whether their RNA genomes are unsegmented or segmented (Koonin et al., 2020) (Figure 1). The unsegmented and some bi-segmented viruses with negative-sense RNA genomes, belong to the order *Mononegavirales* (Koonin et al., 2020; Kuhn et al., 2020). In contrast, most of the segmented viruses with both negative-sense and ambisense RNA genomes belong to the order *Bunyavirales* (Koonin et al., 2020; Kuhn et al., 2020) (Figure 1). Other orders such as *Serpentovirales* (which is the only one of these including NSR phytoviruses), *Muvirales*, *Articulavirales*, among others, have been created to accommodate diverse viruses which have been placed in the major phylogenetic gap between the two large groups of NSR viruses (Wolf et al., 2018).

Twelve genera (*Alphanucleorhabdovirus*, *Betanucleor- habdovirus*, *Coguvirus*, *Cytorhabdovirus*, *Dichorhavirus*, *Emara- virus*, *Gammanucleorhabdovirus*, *Ophiovirus*, *Orthotospovirus*, *Rubodvirus*, *Tenuivirus*, and *Varicosavirus*) belonging to five different families (*Rhabdoviridae, Aspiviridae, Fimoviridae, Tospoviridae*, and *Phenuiviridae*) include species of phytoviruses (Figure 1). Two of the genera (*Coguvirus* and *Rubodvirus*) were recently created to accommodate novel species of NSR viruses related to members of the *Phenuiviridae* family (Navarro et al., 2018b; Diaz-Lara et al., 2019). Furthermore, the ongoing discovery of many novel nucleorhabdoviruses and dichorhaviruses by HTS in the last few years resulted in a split of the genus *Nucleorhabdovirus* into three new genera (Freitas-Astúa et al., 2019). Therefore, as the pace of discovery of new NSR plant viruses using HTS is speedily increasing, the creation of new genera and families to accommodate some of these newly discovered viruses will be a common classification task in future years.

### Rhabdoviridae

The family *Rhabdoviridae* currently comprises 30 genera for 191 species for viruses infectin vertebrates, invertebrates and plants. Six of these genera include 45 species of phytoviruses: *Cytorhabdovirus, Alphanucleorhabdovirus*, *Betanu- cleorhabdovirus* and *Gammanucleorhabdovirus* (unsegmented genomes), and *Dichorhavirus* and *Varicosavirus* (bi-segmented genomes) (Walker et al., 2018; Kuhn et al., 2020) (Figure 1). The virions of these phytorhabdoviruses have bacilliform or rod-shaped morphology, and those with unsegmented genomes are enveloped. Plant-infecting rhabdovirus genomes are 10–16 kb in size and are composed of 6 to 10 genes (Walker et al., 2018) (Figure 1).

The increased application of HTS has seen a significant rise in the number of novel plant rhabdoviruses (Table 1), as well as the completion of genomic sequences of those viruses with poorly characterized genomes (Table 2).

High-throughput sequencing was used successfully to complete the genome sequences of previously reported plant rhabdoviruses where only partial sequence fragments were available, such as Iranian citrus ringspot-associated virus (Sadeghi et al., 2016), ivy vein banding virus (Petrzik, 2012), and soybean blotchy mosaic virus (Lamprecht et al., 2010). Furthermore, HTS could be a key tool to characterize the genomes of some cyto- and nucleorhabdoviruses which have only been characterized biologically, such as broccoli necrotic yellows virus (Lin and Campbell, 1972) and festuca leaf streak virus (Lundsgaard and Albrechtsen, 1976).

Moreover, partial genome fragments of three putative cytorhabdoviruses and one unassigned rhabdovirus were reported, when the viromes of water lily, common bean, *Lamprocephalus* sp. and kalanchoe were analyzed by HTS (Kreuze, 2014; Verdin et al., 2017; Bernardo et al., 2018; Mwaipopo et al., 2018). However, the obtained sequences are not available in any public database, so it is not possible to know if these viruses are novel or may correspond to already known viruses. Thus, it would be useful to apply HTS to complete the genome characterization of these viruses to increase our understanding of plant rhabdovirus diversity. Furthermore, since a tentative nucleorhabdovirus associated with papaya was recently discovered in a papaya virome (Alcalá-Briseño et al., 2020), it would be useful to employ HTS to characterize the molecular properties of papaya apical necrosis virus, a putative nucleorhabdovirus associated with papaya, which was only characterized biologically almost four decades ago (Lastra and Quintero, 1981), to determine if both viruses are related.

#### Cytorhabdovirus

The application of HTS has facilitated the discovery of at least 17 novel cytorhabdoviruses during the last 7 years (Table 1). Furthermore, the use of HTS has allowed characterization of the genomes of five cytorhabdoviruses, for which only a fragment or only the biological properties were known (Table 2). The template mostly used as a source for HTS of these viruses was total RNA, sequenced usually on Illumina platforms (Tables 1, 2).

#### Alpha-, Beta-, Gammanucleorhabdovirus

The application of HTS has facilitated the discovery of ten novel nucleorhabdoviruses during the last 4 years, four alphanucleorhabdoviruses and six betanucleorhabdoviruses (Table 1). Furthermore, the use of HTS has allowed re-sequencing the genomes of two alphanucleorhabdoviruses which had previously been determined using Sanger dideoxy sequencing (maize mosaic virus and maize Iranian mosaic virus). HTS also allowed sequencing of the genome of a betanucleorhabdovirus for which only a genome fragment and the biological properties were known (datura yellow vein virus), and one other for which only the biological properties had been investigated 30 years earlier (sowthistle yellow vein virus) (Table 2). The template mostly used as a source for the HTS was total RNA, sequenced usually with Illumina instruments (Tables 1, 2).

#### Dichorhavirus

The application of HTS has facilitated the discovery of three novel dichorhaviruses during the last 3 years (Table 1). Furthermore, the use of HTS has allowed characterization of the genome of a dichorhavirus for which only a fragment and the biological properties were known (coffee ringspot virus) (Table 2). The template used as a source for the HTS was total RNA, sequenced on Illumina platforms (Tables 1, 2).

#### Varicosavirus

The complete genome of only one varicosavirus, lettuce big-vein associated virus, was previously characterized using Sanger dideoxy sequencing (Sasaya et al., 2002, 2004); however, the application of HTS has facilitated the discovery of two novel varicosaviruses during the last 3 years (Table 1). The template used as a source for the HTS was total RNA and dsRNA, and Illumina and Roche 454 were the technologies employed in the HTS projects (Table 1).

### Fimoviridae

This family is composed of only one genus, *Emaravirus*, for viruses that are distantly related to orthotospoviruses, and exclusively comprise members that have plants as their hosts. Emaraviruses have enveloped, spherical virions, with a diameter of 80–100 nm and a segmented, linear, single-stranded genome with generally four to eight RNA segments (Elbeaino et al., 2018), but a recently described novel emaravirus, perilla mosaic virus, has 10 RNA segments (Kubota et al., 2020).

Most of the members of the genus *Emaravirus* have been recently discovered (Table 1) or characterized in depth by HTS techniques (Table 2). The application of HTS resulted in the discovery of 13 emaraviruses during the last 5 years (Table 1). Different templates, such as total RNA, small RNAs and dsRNA were used as sources for the HTS, and Illumina platforms have been mostly used in the HTS projects (Table 1).

The use of HTS has revealed the genome sequence of two emaraviruses for which only a genomic fragment and some biological properties were known (high plains wheat mosaic virus and pigeonpea sterility mosaic virus 1) (Table 2). Furthermore, the application of HTS has resulted in the detailed characterization of the complete genomes of three emaraviruses. A clearer picture of the genome organization of emaraviruses was obtained by the identification of additional RNA segments. HTS- based research resulted in the identification of three novel genome segments of raspberry leaf blotch virus and rose rosette virus (Di Bello et al., 2015; Lu et al., 2015), and two novel segments for European mountain ash ringspot-associated virus genome (von Bargen et al., 2019). Different templates, including total nucleic acids, total RNA, dsRNA and RNA from partially purified virions were used as sources for the HTS. Illumina platforms were mostly used in these HTS projects (Table 2).

Finally, partial fragments of RNAs1, 2 and 4 of a putative novel emaravirus infecting alfalfa in Australia, named alfalfa ringspot-associated virus were recently identified using HTS (Samarfard et al., 2020). PCR amplification of the conserved termini of the genome segments (Babu et al., 2016) and HTS could be used to characterize the complete genome sequence of this tentative novel emaravirus.

### Aspiviridae

This family, formerly named *Ophioviridae*, contains only one genus, *Ophiovirus*, which is exclusively composed of members that have plants as their hosts (Figure 1). Ophioviruses have non-enveloped, naked filamentous virions and a segmented, linear open circle, serpentine, single-stranded RNA genome, consisting of three to four segments (García et al., 2017).

One novel ophiovirus, blueberry mosaic associated ophiovirus, has been discovered using HTS during the last 6 years (Table 1). Partial genome fragments of three ophioviruses, freesia sneak virus, ranunculus white mottle virus, and tulip mild mottle mosaic virus have been known for some years but their complete genomes remain elusive (García et al., 2017). HTS could be a crucial tool to obtain the complete genomes of these viruses and to expand our understanding of the genomic cues and evolutionary diversity of ophioviruses.

### Tospoviridae

This family contains the single genus *Orthotospovirus*, which is exclusively composed of species for viruses that have plants as their hosts (Figure 1). Orthotospoviruses have enveloped, spherical virions of 80–120 nm diameter and are transmitted by thrips insects in which they also replicate. The genome of orthotospoviruses is segmented with three linear single-stranded RNA segments named large (L), middle (M), and small (S). The L RNA contains one open reading frame in negative-sense polarity, whereas the other two segments, M and S RNAs, are ambisense and have two open reading frames encoding proteins in opposite orientation (Oliver and Whitfield, 2016).

The application of HTS led to the discovery of two novel orthotospoviruses in 2019 and 2020 (Table 1). Total RNA was used as the template and Illumina platforms as the sequencing technology.

The use of HTS has also facilitated completion of the sequence of eight orthotospoviruses for which only one genome segment was available (mostly the S RNA) and their biological properties were known (Table 2). Different templates, including total RNA, small RNA and virus-enriched RNA from purified ribonucleocapsids were used as RNA source, and Illumina platforms were mostly used as sequencing technology in the HTS projects (Table 2).

At the time of this review, only partial genome sequence of four other reported orthotospoviruses are available: groundnut yellow spot virus (S RNA), lisianthus necrotic ringspot virus (S RNA), pepper necrotic spot virus (S RNA) and tomato necrotic ringspot virus (S and M RNAs) (Satyanarayana et al., 1998; Seepiban et al., 2011; Torres et al., 2012; Shimomoto et al., 2014); HTS could enable completion of the genome of these agronomically important viruses.

### Phenuiviridae

Most phenuiviruses have enveloped particles with helical morphology, except tenuiviruses that have non-enveloped filamentous particles, and their genomes are comprised of two to four single-stranded, linear RNA segments. As of 2019, the family *Phenuiviridae* includes 19 ICTV-recognized genera (Figure 1). Only the established genus *Tenuivirus* and likely two recently accepted new genera *Coguvirus* and *Rubodvirus* (2019.026M.A.v1.Phenuiviridae_4gen79sp.xlsx) contain species of plant-infecting viruses (Navarro et al., 2018b; Diaz-Lara et al., 2019). Phylogenetic relationships with other phenuiviruses support their classification within this family (Diaz-Lara et al., 2019).

#### Coguvirus

This genus is composed of two previously unknown viruses that have bi-segmented genomes, which have been discovered using HTS of citrus (Navarro et al., 2018a,b), while two tentative coguviruses that have tri-segmented genomes, have been discovered using HTS in watermelon (Xin et al., 2017) (Table 1). Total RNA and small RNAs were used as template sources for HTS on Illumina platforms (Table 1). Recently, the complete tri-segmented genomes of four cogu-like viruses were determined when the virome of grapevine was characterized (Bertazzon et al., 2020; Chiapello et al., 2020) (Table 1).

Moreover, partial segments of a cogu-like virus were discovered when the virome of ornamental flowers was analyzed by HTS (Wylie et al., 2019). However, it will be necessary to complete the genome characterization and perform further analyses of these viruses to confirm if they are in fact plant-infecting viruses or arthropod-infecting viruses whose RNA was co-purified along with the plant RNA. This additional information would enable further characterization of these putative coguviruses, thus increasing our understanding of the plant-infecting phenuivirus diversity and evolutionary history.

#### Rubodvirus

This genus is composed of species for two previously unknown viruses that have tri-segmented genomes which have been discovered using HTS in apple (Rott et al., 2018; Wright et al., 2018), while two tentative rubodviruses were discovered using HTS in grapevine (Diaz-Lara et al., 2019) (Table 1). DsRNA and total RNA were the templates used as sources for HTS on Illumina platforms (Table 1).

#### Tenuivirus

Tenuiviruses have a segmented genome of four to eight single-stranded RNA segments with negative-sense or ambisense polarity (Gaafar et al., 2019a). The application of HTS led to the discovery of two new tenuiviruses, one infecting melon and black medic (Gaafar et al., 2019a; Lecoq et al., 2019) and the other from sugarcane associated with Ramu stunt disease (Mollov et al., 2016) (Table 1). In addition, a recent HTS study resulted in the molecular characterization of a wheat tenuivirus, the biological properties of which had been previously characterized in the 1960s (Sõmera et al., 2020) (Table 1). Total RNA and small RNAs were used as sources for HTS on Illumina platforms (Table 1). Moreover, the use of HTS facilitated sequencing of a tenuivirus from rice, for which only the sequence of RNA4 and its biological properties were known previously (Table 2). At the time of writing this review, only partial genomic sequence of four other tenuiviruses are known: maize stripe virus (complete sequences of RNAs 2, 3, 4 and 5), maize yellow stripe virus (partial sequences of RNAs 1, 2, 3, 4 and complete sequence of RNA 5), Iranian wheat stripe virus (complete sequences of RNAs 2, 3 and 4), wheat yellow head virus (complete sequence of the nucleoprotein gene) (Huiet et al., 1991; Estabrook et al., 1996; Seifers et al., 2005; Heydarnejad et al., 2006; Mahmoud et al., 2007). HTS would be an essential tool to obtain the complete genomes of these viruses and expand our knowledge of genomic architecture of tenuiviruses.

## Conclusion and Perspectives

The number of NSR plant viruses identified so far represents a negligible fraction of the potential number of NSR viruses present in the virosphere. The discovery and in-depth molecular characterization of these viruses has been challenging given their extensive divergence, their outstanding diversity in terms of genomic architecture, and mostly, the negligible share of plant species studied for virus discovery. However, the widespread implementation of HTS, a cost-effective and efficient technology platform, allowed the discovery and molecular characterization of the genome sequences of at least 70 NSR plant viruses in the last few years. Thus, we predict that the increasing use of HTS, not only for plant samples but also in arthropod vectors, will allow the identification of many novel NSR phytoviruses which will be crucial to unravel the evolutionary landscapes of many NSR virus clades that are poorly characterized today. Nevertheless, as many more novel NSR viruses are being discovered, careful analysis will be essential to confirm their correct ecological context, to determine whether the new viral agents are plant-infecting viruses, fungi- or arthropod-infecting viruses whose RNA was co-purified along with the plant RNA, or just contamination-inadvertently sampled environmental viruses.

As soon as the virome characterization of multiple samples of cultivated and wild plants becomes more routine, more viruses with unique features and novel phylogenetic relationships will likely be discovered. As we assess the viral ‘dark matter,’ we will start to grasp the evolutionary history of plant viruses, eventually leading us to untangle the diversity of the NSR virosphere and gain increased knowledge about its complex evolutionary and phylogenetic relationships. A rich picture of the plant virome landscape will also provide an essential framework for improved taxonomic classification of plant NSR viruses as part of the realm *Riboviria*. In conclusion, HTS-based methods for virus discovery, our ‘new eyes,’ are unraveling in real time the richness and magnitude of the plant RNA virosphere.

## Author Contributions

All authors wrote the manuscript and approved it for publication.

## Conflict of Interest

The authors declare that the research was conducted in the absence of any commercial or financial relationships that could be construed as a potential conflict of interest.
